# Patients with type 2 diabetes mellitus present similar immunological response to COVID-19 BNT162b2 mRNA vaccine to healthy subjects: a prospective cohort study

**DOI:** 10.1007/s42000-022-00405-7

**Published:** 2022-10-21

**Authors:** Stavroula A. Paschou, Vangelis Karalis, Theodora Psaltopoulou, Ioanna Charitaki, Aimilia D. Sklirou, Vassiliki A. Iconomidou, Vasiliki Vasileiou, Georgia N. Kassi, Andromachi Vryonidou, Alexander Kokkinos, Nicholas Tentolouris, Erifili Hatziaggelaki, Ioannis P. Trougakos, Evangelos Terpos, Meletios Athanasios Dimopoulos

**Affiliations:** 1 Endocrine Unit and Diabetes Center, Department of Clinical Therapeutics, Alexandra Hospital, School of Medicine, National and Kapodistrian University of Athens, Athens, Greece; 2 Faculty of Pharmacy, School of Health Sciences, National and Kapodistrian University of Athens, Athens, Greece; 3 Department of Clinical Therapeutics, Alexandra Hospital, School of Medicine, National and Kapodistrian University of Athens, 80 Vasilisis Sophias Ave, 11528 Athens, Greece; 4 Department of Cell Biology and Biophysics, Faculty of Biology, National and Kapodistrian University of Athens, Athens, Greece; 5 Department of Endocrinology, Alexandra Hospital, Athens, Greece; 6 Department of Endocrinology and Diabetes Center, Hellenic Red Cross Hospital, Athens, Greece; 7 Diabetes Center, First Department of Propaedeutic Internal Medicine, Laiko General Hospital, National and Kapodistrian University of Athens, Athens, Greece; 8 Diabetes Center, Second Department of Internal Medicine, Attikon University Hospital, National and Kapodistrian University of Athens, Athens, Greece

**Keywords:** Type 2 diabetes, Diabetes mellitus, Hyperglycemia, Vaccination, COVID-19, Immune response, SARS-CoV-2

## Abstract

**Aim:**

To compare the kinetics of neutralizing antibodies (NΑbs) against SARS-CoV-2 after vaccination with the BNT162b2 mRNA vaccine (Comirnaty, Pfizer/BioNTech) between patients with T2DM and healthy controls.

**Methods:**

NAb levels after the BNT162b2 mRNA vaccine were compared between 50 patients with non-insulin treated T2DM and 50 age-, gender-, and BMI-matched healthy controls up to 3 months after the second dose. The median age of both groups was 70 years.

**Results:**

On day 1, mean NAbs of the control and T2DM groups were 14.64% (standard error, SE = 2.30) and 14.04% (SE = 2.14), respectively (*p* value = 0.926). Three weeks later, the mean NAb values were 39.98% (SE = 3.53) in the control group and 40.97% (SE = 3.99) in participants with T2DM (*p* value = 0.698). One month after the second vaccination, mean NAb values increased to 87.13% (SE = 2.94) in the control group and 89.00% (SE = 2.18) in the T2DM group. Three months after the second vaccine dose, the mean inhibitory titers decreased to 83.49% (SE = 3.82) (control group) and 76.36% (SE = 3.33) (T2DM group). On all occasions, no significant difference was found between the two groups (all *p* values > 0.05).

**Conclusions:**

Patients with T2DM present similar immunological response to COVID-19 BNT162b2 mRNA vaccine to that of healthy subjects.

## Introduction

The prevalence of type 2 diabetes mellitus (T2DM) is around 10% in Western countries, reaching, in fact, epidemic proportions. This phenomenon is broadly associated with aging and obesity [1]. Since the beginning of the SARS-CoV-2 pandemic, several studies have consistently indicated high prevalence of DM (around 20%) in patients with COVID-19 who need hospitalization [2, 3]. Moreover, DM is even more frequent, up to 35%, in patients who experience severe COVID-19 and need oxygen or treatment in intensive care units (ICUs) [2–4]. The mortality rates in the DM population have also been very high, around 25% in most cohorts [5, 6]. Therefore, patients with DM are considered to be at high risk for COVID-19 adverse outcomes and mortality.

Vaccination against SARS-CoV-2 is the most powerful and promising tool against the pandemic. As patients with DM are at high risk for severe disease, vaccination is highly recommended in this population, which has been prioritized in the vaccination strategies of most countries. Endocrine and diabetes societies worldwide reported early on that vaccines against COVID-19 are safe and recommended that all patients with endocrine disease should be vaccinated, including those with DM [7, 8]. However, several questions have been raised about the efficacy of vaccines in patients with existing medical problems, including those with DM. If a compromised immune response to SARS-CoV-2 is probably a reason for the increased risk for severe COVID-19 in patients with DM, the question of whether patients with DM also have an impaired immunological response to vaccination is crucial [4].

Major studies for the approval of vaccines against COVID-19 included high percentages of participants with various metabolic problems, such as obesity, T2DM, hypertension, and dyslipidemia. Phase III studies on both mRNA and traditional vaccines did not report any differences in possible side effects or immune response characteristics in patients with DM, although not many details were given [9–11]. Two Italian studies including health workers vaccinated with the mRNA Pfizer/BioNTech vaccine provided evidence that obesity, as indicated by higher body mass index (BMI) or central obesity, as indicated by increased waist circumference (WC), may be associated with weaker antibody titers [12, 13]. These results raised some further concerns about the efficacy of vaccines in patients with metabolic problems in general. A subsequent large study from Israel suggested the presence of lower antibody levels in response to the mRNA Pfizer/BioNTech vaccine in a specific sub-group of participants with DM. However, the number of patients with DM was low, and mainly IgA antibody titers were lower in this group [14]. A recent multicenter study from Austria, including both T1DM and T2DM patients after mRNA Moderna (96.5%) or mRNA Pfizer/BioNTech (3.5%) vaccination, showed similar immunological responses for all patients and controls groups independently of the level of the glycemic control [15].

As the topic remains quite controversial, we aimed to compare immune responses, namely, the kinetics of neutralizing antibodies (NAbs), against SARS-CoV-2 after vaccination with BNT162b2 mRNA vaccine (Comirnaty, Pfizer/BioNTech) between patients with T2DM and healthy subjects.

## Patients and methods

### Clinical setting

A single-center prospective cohort study was conducted at the Department of Clinical Therapeutics, Alexandra Hospital, School of Medicine, National and Kapodistrian University of Athens, Greece, after approval from the relevant Ethical Committee. The main inclusion criteria were age older than 18 years and the ability to sign an informed consent form. Active malignancies, use of immunosuppressive drugs, and end-stage renal failure were the primary exclusion criteria. Two groups of subjects were included in this study, namely, non-insulin treated patients with T2DM and healthy subjects. Baseline demographics were related to age, gender, BMI, and concomitant diseases. To account for the potentially confounding effects of covariates, case–control matching was performed to match the two groups for age, gender, and BMI.

The Declaration of Helsinki and the International Conference on Harmonization of Good Clinical Practice were followed throughout the clinical part. Informed consent was given by all participants before participation in the study. In accordance with the General Data Protection Regulation, participants’ data were kept confidential. All identities were removed to maintain anonymity. To avoid patient identification, names were deleted immediately after recruitment and replaced with a random number.

### Measurement of NAbs

Blood was drawn from the T2DM and control groups on the first day of vaccination, on day 22 (before the second dose), and at 1 and 3 months after the second dose. Within 4 h after blood collection, serum was isolated and stored at 80 °C until the day of measurement. At the above time points, NAbs against SARS-CoV-2 were quantified using an FDA-approved method (ELISA, cPassTM SARS-CoV-2 NAbs Detection Kit; GenScript, Piscataway, NJ, USA). Samples from the same patient or control were measured in the same ELISA plate.

### Statistical analysis

First, descriptive statistical criteria and scatter metrics were calculated. A normality test was performed before statistical comparison between groups. To determine the normality of data distribution, the Shapiro–Wilk test was applied. The independent *t* test was used to compare the two different groups (e.g., control group vs. T2DM group). When comparing the same group of subjects at subsequent time points, the repeated measures ANOVA method was used. If statistical significance was found, the Bonferroni post hoc test was used to determine which specific means were different. All statistical comparisons were two-sided, and the significance level was set at 5%. A result was considered significant if the estimated *p* value (*p*) was below the significance level. All statistical analyses were performed in Python v.3.9.2.

## Results

### Characteristics of the participants

The BNT162b2 mRNA vaccine was administered in two doses to 50 healthy subjects and 50 patients with non-insulin treated T2DM. The demographic data of the study participants are summarized in Table 1*.* The T2DM group included 21 men (42%) and 29 women (58%), whereas the control group had a similar proportion of subjects, 22 men (44%) and 28 women (56%). The median age of both groups was 70 years, and the median BMI value was 29.7 kg/m^2^ and 27.6 kg/m^2^ for the T2DM and control group, respectively.

**Table 1:** Characteristics of study participants

Characteristics	T2DM group	Control group
Sample size	50	50
Gender		
Men	21 (42%)	22 (44%)
Women	29 (58%)	28 (56%)
Age (median) [years]	70	70
BMI (median) [kg/m^2^]	29.7	27.6
Underweight or normal weight (*n*, %)	10 (20%)	10 (20%)
Overweight or obese (*n*, %)	40 (80%)	40 (80%)

*T2DM*, type 2 diabetes mellitus; *BMI*, body mass index; *n*, number of subjects

### NAbs

**Fig. 1:**
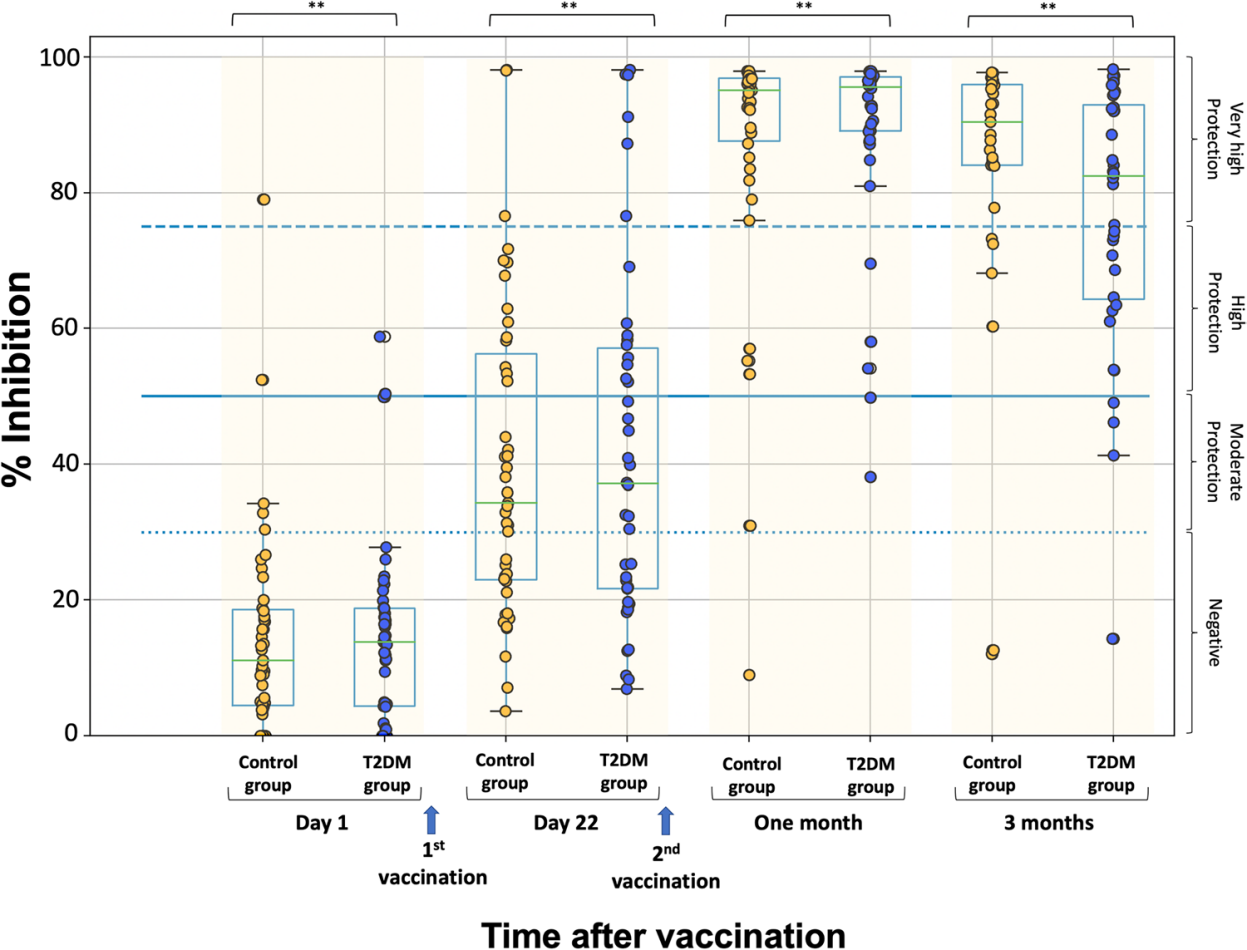
Inhibition (%) of SARS-CoV-2 binding to the human host receptor angiotensin-converting enzyme in healthy and T2DM subjects on the day of the first vaccination (day 1), on the day of the second vaccination (day 22), 1 month later, and 3 months after the second dose. The boxplot boundaries represent the quartiles of the distribution, while the overlaid points represent the individual NAb inhibition values. The dashed lines reflect the levels of inhibition, which are 30, 50, and 75%. The independent *t* test was used to compare the two groups (i.e., control group vs. T2DM group). When comparing the same group of subjects at subsequent time points, the repeated measures ANOVA method was used. The double asterisk (**) indicates statistically significant differences (*p* value < 0.05) in NAb values between the different measurement days

Figure 1 presents the percent inhibition of NAbs on the day of the first dose (just before vaccination), on the day of the second dose (i.e., 21 days later and just before vaccination), 1 month later (i.e., day 50 after the start of the study), and 3 months after the second vaccination dose. On day 1, the mean NAbs of the control and T2DM groups were 14.64% (standard error (SE) = 2.30) and 14.04% (SE = 2.14), respectively (*p* value = 0.926), indicating that participants in both groups started from the same baseline values. Three weeks later, just before the second vaccination dose, the mean NAb values were 39.98% (SE = 3.53) in the control group and 40.97% (SE = 3.99) in participants with T2DM (*p* value = 0.698). One month after the second vaccination, mean NAb values had increased to 87.13% (SE = 2.94) in the control group and 89.00% (SE = 2.18) in the T2DM group. Three months after the second vaccine dose, the mean inhibitory titers had decreased to 83.49% (SE = 3.82) (control group) and 76.36% (SE = 3.33) (T2DM group). It is worth noting that 3 months after the second vaccination, 48 subjects (96%) in the control group and 46 patients with T2DM (92%) still had high or very high titers. On all occasions, no significant difference was found between the two groups (all *p* values > 0.05).

To further investigate the possible effects of T2DM on the immune response by vaccination, the activity of NAbs was examined separately for males and females. The overall profiles are similar to those described above for the entire study population. On day 1, NAb activity was similar in males and females in the T2DM group and the control group (*p* values of 0.618 and 0.553 for males and females, respectively). In male participants, inhibition levels were similar at all subsequent time points and followed the general trend described above. Similar results were observed in females, with one difference only, namely, that 3 months after the second vaccination, a statistical difference was observed between NAb titers of T2DM and those of healthy subjects (*p* value = 0.008).

The role of age and BMI in the immune response of T2DM subjects compared to healthy subjects was further investigated. We did not find any significant differences between T2DM and controls at the various time points. It should be noted that younger patients (40 to 70 years of age) with T2DM tend to have a variable response toward a more rapid elimination of NAbs from the body compared to controls. Moreover, BMI does not seem to influence the immune response both in T2DM and control groups.

## Discussion

No differences in the immunological response after vaccination with the BNT162b2 mRNA vaccine (Comirnaty, Pfizer/BioNTech) between patients with T2DM and healthy subjects, specifically on the day of the second dose, 1 month later, and 3 months after the second dose, were observed in the current study.

Participants in both groups started from the same baseline NAb values. Three weeks later, just before the second vaccination dose, the mean NAb values were 39.98% in the control group and 40.97% in participants with T2DM. One month after the second vaccination, mean NAb values increased to 87.13% in the control group and 89.00% in the T2DM group. Three months after the second vaccine dose, the mean inhibitory titers decreased to 83.49% (control subjects) and 76.36% (T2DM). It is worth noting that 3 months after the second vaccination, a large percentage of both patients and controls had still high or very high titers.

These findings are of great significance as T2DM is today a very common clinical problem worldwide, affecting around 10% of the adult population in Western countries [1]. Most importantly, T2DM patients are considered to be at high risk for COVID-19 adverse outcomes and mortality [2–6]. Indeed, DM is commonly found in patients with COVID-19 who need hospitalization (around 20%) and in those who experience severe COVID-19 and need oxygen or treatment in the ICU (up to 35%) [2, 3]. The mortality rates for DM patients have also been very high (around 25%). Therefore, vaccination, which is the most powerful tool against the pandemic, is highly recommended in this population [5, 6]. The results of this study confirm the efficacy of the BNT162b2 mRNA vaccine (Comirnaty, Pfizer/BioNTech) in patients with T2DM. The clinical significance is high, as a compromised immune response which leads to adverse outcomes after SARS-CoV-2 infection is likely to indicate an impaired immunological response to vaccination as well.

Our results are in accordance with a few previous studies. Firstly, major studies for the approval of vaccines against COVID-19 included participants with T2DM. These studies, with both mRNA and traditional vaccines, did not report any differences in patients with DM [9–11]. A recent multicenter study from Austria, including both T1DM and T2DM patients after mRNA Moderna (96.5%) or mRNA Pfizer/BioNTech (3.5%) vaccination, showed similar immunological response for all patients and control groups. Anti-spike IgG antibodies were measured in this study. Interestingly, the results were independent of the level of the glycemic control [15]. A prospective observational study from Greece examined IgG antibody titers in participants with and without DM after vaccination with the mRNA Pfizer/BioNTech vaccine. Almost 17% of participants with DM did not develop adequate humoral immune response after the first dose; however, it was observed to be high and similar after the second dose in participants both with and without DM and remained so almost 2 months after the second dose of the vaccine [16].

On the other hand, a study from Kuwait evaluated the levels of both anti-SARS-CoV-2 IgG and NAbs in patients with T2DM and/or other metabolic risk factors (namely, hypertension and obesity) compared to those without after two doses of the mRNA Pfizer/BioNTech vaccine. Both T2DM and non-diabetic individuals displayed a robust response to vaccination, as demonstrated by their high antibody titers. However, both SARS-CoV-2 IgG and NAb titers were lower in subjects with T2DM [17]. A large study from Israel also pointed to lower antibody levels in response to the mRNA Pfizer/BioNTech vaccine for a specific sub-group of participants with DM. It is however worth mentioning that mainly IgA antibody titers were lower in the DM group in this study [14]. A major strength of the current study is the measurement of NAbs, which represents the type of antibodies that are best able to neutralize or to effectively defend against the virus. They are considered the most representative type of functional antibodies, providing both quantitative and qualitative information regarding the immune response profile [18–20].

Patients and controls were gender-matched; however, we investigated the impact of this parameter on the immunological response. No significant differences were seen in the comparisons between men and women for the control group, T2DM, and the total cohort separately. Comparing the activity of NAbs between males and females of the T2DM and control groups, we found overall profiles similar in men to those described regarding the entire study population. Women also exhibited no differences at most time points, with the exception of 3 months after the second vaccination. At this time point, median inhibition levels were lower in the female T2DM group than in the control female group. There are distinct gender differences in metabolism regulation, including insulin sensitivity, while hormonal differences between the two genders can affect their response to stress and the inflammatory processes that follow. Interestingly, SARS-CoV-2 binding or proliferation can potentially be affected at a cellular or molecular level of gender genetic and hormonal differences [21].

Regarding the possible role of age in NAb values, we did not find any significant differences between T2DM and controls at the various time points. It is notable that younger patients (40 to 70 years of age) with T2DM tend to have a variable response toward a more rapid elimination of NAbs from the body compared to controls. We have previously reported a negative effect of age on immunological response, which gradually decreases as we move away from the day of vaccination [22, 23]. This is also implied in the current study, although sound relevant conclusions cannot be reached. Patients and controls were age-matched in the current study; however, the median age of both groups was high (70 years), and a distribution from low values (i.e., just below 40 years) to high values (i.e., above 90 years) can be observed. Indeed, the most frequent age estimates were just below 65 years and slightly above 80 years. Aging patients with T2DM present several specific characteristics and treatment needs, especially in this era of the COVID-19 pandemic [24].

Moreover, BMI does not seem to influence the immune response either in the T2DM group or in the control group. Major studies for the approval of vaccines against COVID-19 included large percentages of participants with obesity and did not report any differences in immune response. However, two Italian studies including health workers vaccinated with the mRNA Pfizer/BioNTech vaccine provided evidence that obesity, as indicated by BMI values, or central obesity, as reflected by increased WC, may be associated with weaker antibody titers [12, 13]. These results raised concern about the efficacy of vaccines based on BMI, but they are not confirmed by the current study.

A major strength of this study is the measurement of specific NAbs, as these are considered the most representative type of functional antibodies. Furthermore, we specifically studied non-insulin treated patients with T2DM in order to have a homogeneous patient population and to avoid biases due to glycemic and other metabolic parameters. A limitation of the study from an epidemiological point of view might be the relatively small sample size. Moreover, although patients and controls were age-matched, the increased median age could potentially influence the results regarding the role of age in the immune response. Lastly, a number of clinical, biochemical, and disease characteristics of the patients were not available.

In conclusion, this study provided evidence that patients with T2DM present similar immunological response to the COVID-19 BNT162b2 mRNA vaccine (Comirnaty, Pfizer/BioNTech) to healthy subjects.
